# Intra-aortic balloon pump-assisted major hepatectomy in a case with coronary disease

**DOI:** 10.1186/s40792-015-0114-5

**Published:** 2015-11-02

**Authors:** Kazuo Okadome, Hiromitsu Hayashi, Takaaki Higashi, Hideaki Takeyama, Keita Sakamoto, Hideyuki Kuroki, Hidetoshi Nitta, Daisuke Hashimoto, Akira Chikamoto, Toru Beppu, Hideo Baba

**Affiliations:** Department of Gastroenterological Surgery, Graduate School of Life Sciences, Kumamoto University, 1-1-1 Honjo, Kumamoto, 860-8556 Japan

**Keywords:** Major hepatectomy, IABP, Coronary disease

## Abstract

Clinically, we often encounter cancer patients who also have cardiovascular disease such as coronary artery disease. We experienced a case of severe coronary artery disease and a large hepatocellular carcinoma in a 69-year-old man. To reduce the risk of a perioperative cardiovascular event during mesohepatectomy, an elective intra-aortic balloon pump (IABP) was used. After an uneventful recovery, the patient was discharged on day 15. While IABP is frequently introduced in cardiac surgery, there have been few reports of its use during liver surgery. Here, we present IABP-assisted major hepatectomy as an option in a patient with both cancer and coronary artery disease.

## Background

Although advances in multimodal treatment options, such as radiofrequency ablation, interventional radiology, and chemotherapy, have improved the rate of survival in patients with hepatocellular carcinoma (HCC), hepatic resection still provides the best chance of a cure, especially in patients with HCC that has progressed beyond the Milan criteria. In a randomized control study comparing hepatectomy and trans-arterial chemoembolization (TACE) for resectable HCC that had progressed beyond the Milan criteria, better survival outcomes were achieved with hepatectomy [1].

Because older age is a risk factor for HCC [2], the number of elderly patients requiring hepatectomy has increased worldwide [3]. Similarly, the likelihood of developing cardiovascular disease increases with age, resulting in a greater number of HCC patients with cardiovascular disease. In patients with severe coronary disease who undergo percutaneous coronary intervention (PCI) using a drug-eluting stent (DES), oral administration of dual antiplatelet agents is required for at least 6–12 months after the PCI [4]. However, owing to the risk of considerable perioperative blood loss with oral antiplatelet agents, curative hepatectomy is contraindicated. Furthermore, major hepatectomy is still associated with significant morbidity and mortality [5]. As an optional strategy, we present a case with a large HCC and severe coronary artery disease which was successfully treated with an intra-aortic balloon pump (IABP)-assisted mesohepatectomy.

## Case presentation

A 69-year-old man was admitted for treatment of a large, 12-cm hepatic tumor. He had chronic hepatitis C with a sustained virologic response to interferon treatment. Plain computed tomography (CT) showed a heterogeneous, hypodense tumor located in segments IV/V/VIII of the liver. The tumor showed contrast enhancement and washout in the arterial and late phases. The levels of alpha-fetoprotein, *Lens culinaris* agglutinin-reactive fraction of alpha-fetoprotein, and protein induced by vitamin K absence/antagonist-II were 13.7 ng/mL, <0.5 %, and 14,699 mAU/mL, respectively. The indocyanine green retention rate at 15 min was 6.6 %. The patient was classified as having Child-Pugh class A liver function based on the following parameters: total bilirubin, 0.9 mg/dL; albumin, 4.1 g/dL; prothrombin activity, 91 %; ascites, absent; and hepatic encephalopathy, absent. The ratio of 99mTc-galactosyl human serum albumin (GSA) scintigraphy uptake by the liver to that by the liver plus heart at 15 min was 0.97. Curative hepatectomy, in the form of mesohepatectomy, was initially planned.

**Fig. 1:**
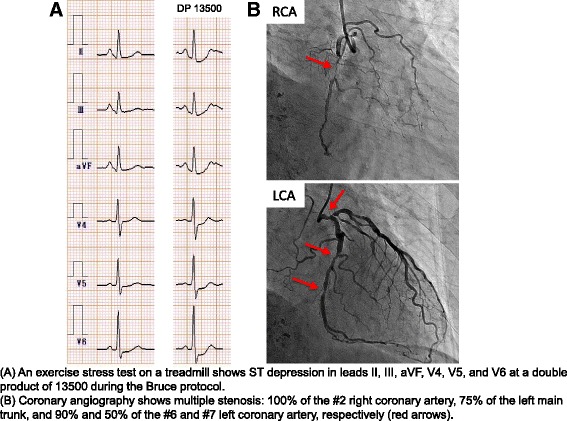
Asymptomatic myocardial ischemia. **a** An exercise stress test on a treadmill shows ST depression in leads II, III, aVF, V4, V5, and V6 at a double product of 13,500 during the Bruce protocol. **b** Coronary angiography shows multiple stenosis: 100 % of the #2 right coronary artery, 75 % of the left main trunk, and 90 and 50 % of the #6 and #7 left coronary arteries, respectively (*red arrows*)

However, an exercise stress test on a treadmill showed ST depression in leads II, III, aVF, V4, V5, and V6 at a double product of 13,500 in the Bruce protocol (Fig. 1) despite no apparent findings on the electrocardiogram and echocardiogram. Then, coronary contrast CT and 201Tl myocardium scintigraphy revealed asymptomatic myocardial ischemia (inferioseptal ischemia). During coronary angiography, multiple stenoses were detected: 100 % of the #2 right coronary artery, 75 % of the left main trunk, and 90 and 50 % of the #6 and #7 left coronary arteries, respectively (Fig. 1). A multidisciplinary discussion between the hepatic surgery, cardiovascular surgery, and cardiovascular medicine departments was conducted. The use of a bare metal stent in the left main trunk is unreliable, a DES requires oral administration of a dual antiplatelet agent for at least 6–12 months, and coronary artery bypass grafting (CABG) can be conducted 1 month after hepatectomy if everything goes smoothly [4, 6]. However, the risks with mesohepatectomy are high in the presence of oral anticoagulant and antiplatelet administration. Therefore, as an initial therapeutic strategy, the following sequential treatments were planned: TACE for local disease control, CABG, and mesohepatectomy without the use of an anticoagulant or antiplatelet agent.

**Fig. 2:**
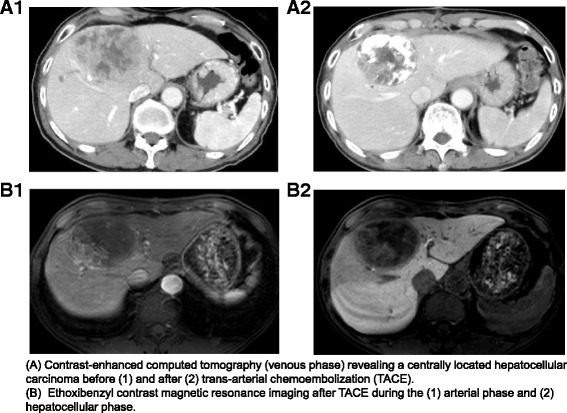
Hepatocellular carcinoma. **a** Contrast-enhanced computed tomography (venous phase) revealing a centrally located hepatocellular carcinoma before (1) and after (2) trans-arterial chemoembolization (TACE). **b** Ethoxibenzyl contrast magnetic resonance imaging after TACE during the (1) arterial phase and (2) hepatocellular phase

One month following successful TACE to prevent aggressive disease progression, CABG (left internal thoracic artery-left anterior descending coronary artery [LITA-LAD], aorta-saphenous venous graft-right coronary artery [AO-SVG-RCA]) was performed. However, the coronary three-dimensional CT on day 7 post-CABG showed a poorly described LITA-LAD bypass, and myocardial scintigraphy revealed a re-distributed image in the middle reserve on the apex side, indicating remaining myocardial ischemia. Although the HCC was partially controlled by TACE, partial viability remained (Fig. 2). Therefore, we finally decided to perform an IABP-assisted mesohepatectomy as curative treatment for HCC.

**Fig. 3:**
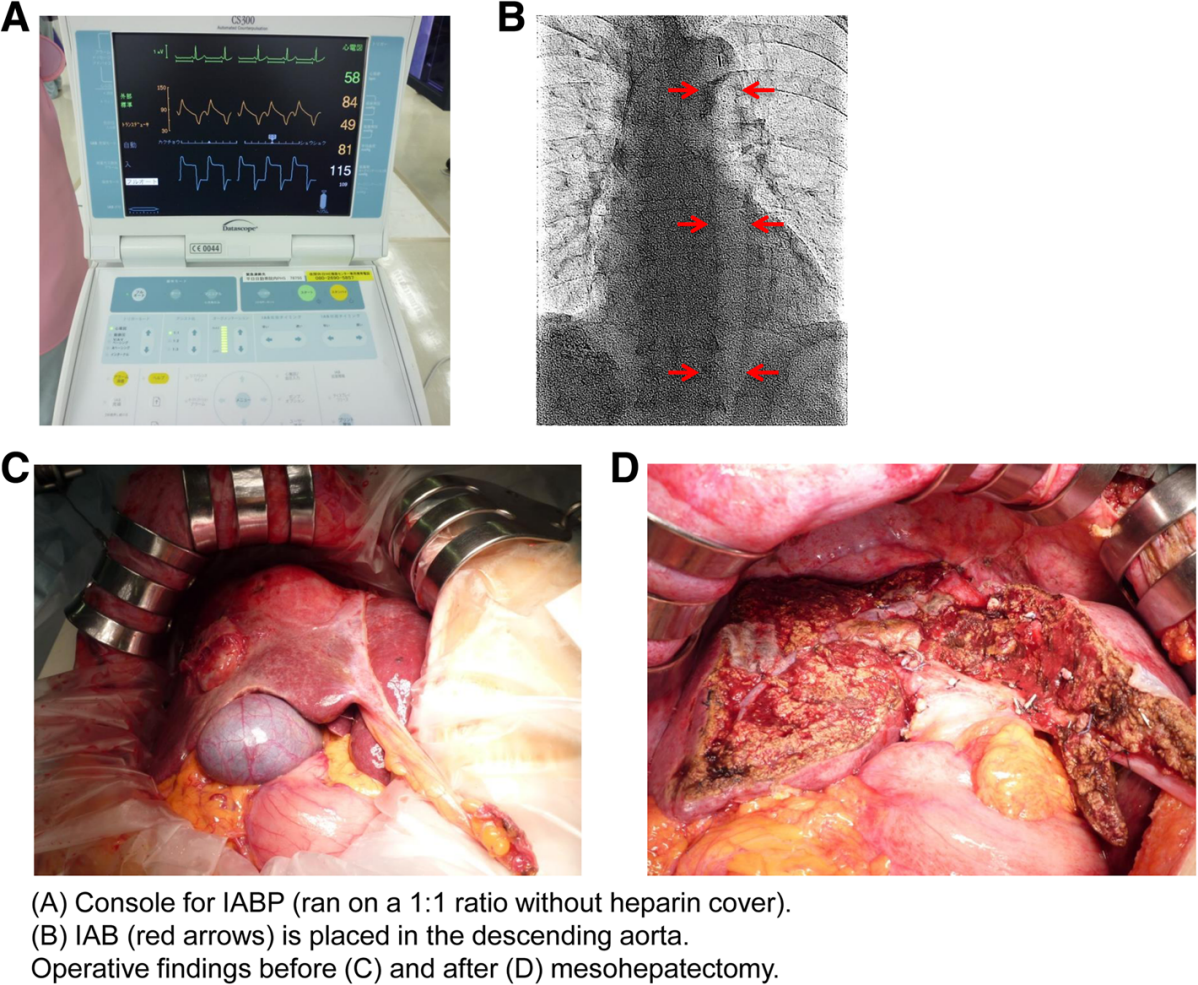
Details of intra-aortic balloon pump (IABP)-assisted mesohepatectomy. **a** Console for IABP (ran on a 1:1 ratio without heparin cover). **b** IAB (*arrows*) is placed in the descending aorta. Operative findings before (**c**) and after (**d**) mesohepatectomy

Eleven weeks after the TACE, the IABP-assisted mesohepatectomy was performed (Fig. 3). The antiplatelet (aspirin) and anticoagulant (warfarin potassium) agents were stopped 9 and 7 days, respectively, before the hepatectomy. Heparin was continuously and intravenously administered for 6 h before the hepatectomy. First, the IAB was inserted from the left femoral artery under local anesthesia and perioperatively ran at a 1:1 ratio without a heparin cover. To avoid a cardiovascular event, intravenous nicorandil and isosorbide mononitrate were continuously administered. There was no apparent change in vital signs after the IABP was inserted. Conventional liver resection was performed through an exclusive abdominal incision. Liver transection was performed using the Pean clamp-crushing technique and TissueLink (TissueLink Medical Inc., Dover, DE) or a VIO soft coagulation system (ERBE, Elektromedizin GmbH, Germany). The Pringle maneuver or hemihepatic vascular occlusion was applied during parenchymal transection. In addition to the general vital signs, hemodynamics were monitored using a FloTrac Sensor®, and transesophageal echocardiography was monitored; the patient remained stable during the operation. During the mesohepatectomy, 998 mL blood was lost with transfusion of 6 units red cell concentrate, and the operative time was 5 h and 31 min. The IABP was removed the following morning without any problem.

Standard systemic antibiotic therapy using cefmetazole was routinely administered immediately before surgery and then twice daily for the first three postoperative days. Nicorandil and isosorbide mononitrate were also administered after surgery, and the route of administration was changed to oral and transdermal patches, respectively, on postoperative day 3. The antiplatelet agent (aspirin) was also resumed on postoperative day 3 without any sign of postoperative bleeding. The postoperative course was uneventful, and he was discharged on postoperative day 15 (Clavien-Dindo classification, grade 1). The liver tumor was pathologically diagnosed as poorly differentiated HCC. Then, PCI was successfully performed.

## Conclusions

IABP is the most common mechanical circulatory assistance device for a variety of clinical conditions with cardiovascular disease. Since the introduction of the counterpulsation concept in the 1960s, the indications for its use have expanded [7] and include acute myocardial infarction, cardiogenic shock, high-risk coronary intervention, and surgery for high-risk cardiovascular patients [8]. IABP improves myocardial perfusion and reduces afterload. Balloon inflation during diastole increases diastolic pressure, which improves myocardial perfusion, because coronary perfusion occurs mainly during diastole. In addition, balloon deflation during systole decreases systolic blood pressure, reducing the afterload and therefore the myocardial oxygen demand [8, 9].

The elective use of IABP in high-risk patients (e.g., those with preoperative left ventricular ejection fraction ≤40 %, left main coronary artery stenosis ≥70 %, reoperation, and/or unstable angina despite medical treatment) prior to high-risk CABG has been the most widely investigated [10]. Although the indications for the prophylactic use of IABP for high cardiac risk non-cardiac surgery have not been determined, the following categories were suggested as high cardiac risk non-cardiac surgery in 1991: Goldman class III or IV; New York Heart Association class III or IV for angina; unstable angina; angiographic evidence of multivessel or left main coronary artery disease in conjunction with evidence of ischemia with exercise or dipyridamole-thallium scintigraphy; or angiographically documented multivessel coronary artery disease with a history of hemodynamic instability during previous non-cardiac surgery [11]. The successful use of prophylactic IABP and decision analysis has also been reported [12], with the most benefit with IABP-assisted surgery occurring for major surgical procedures in patients at a high risk of postoperative complications (Goldman class IV or Detsky class III).

However, in the present case, myocardial ischemia remained in the middle of the apex side despite the CABG (LITA-LAD, AO-SVG-RCA), indicating that the LITA-LAD bypass was not successful; stenosis in 75 % of the left main trunk and 90 % of #6 and 50 % of #7 in the left anterior descending coronary artery remained. Despite the Goldman class I and Detsky class I classifications, we thought that the use of IABP was beneficial during the hepatectomy. Although the rates of morbidity and mortality have decreased with hepatectomy, high morbidity and mortality rates remain, particularly with mesohepatectomy [5, 13]. For example, the 30-day mortality and major morbidity rates were reportedly 2.5 and 19.6 %, respectively, in 2313 hepatectomies in the National Surgical Quality Improvement Program dataset [14], and the major morbidity and mortality rates reflected the extent of hepatic resection.

Regarding cardiac risk, hepatectomy was considered high risk based on a cardiac event (cardiac death and myocardial infarction) in the 2014 European Society of Cardiology/European Society of Anaesthesiology Guidelines [15]. The risks of ischemic outcomes (death/myocardial infarction) and primary bleeding were 3.1 and 1.3 %, respectively, with surgical procedures 30 days after CABG in the Study in Kyoto PCI/CABG Registry Cohort-2 [16].

Although the use of IABP has been associated with complications, an analysis of data from a registry of 16,909 persons who underwent a procedure with IABP between 1996 and 2000 reported that major IABP-related complications (major limb ischemia, severe bleeding, balloon leak, or death due directly to IABP insertion or failure) occurred in only 2.6 % of the patients [17]. Moreover, in the 30 published cases of non-cardiac surgeries with IABP, all with high-risk patients with coronary disease, the outcomes were favorable, and no death was attributable to the IABP [10, 11, 18–25]. Because the rate of complications with IABP are likely decreasing with improvements in technique, equipment, and experience, the criteria suggested by Georgeson et al. in 1992 could expand further [12].

There have been few studies to investigate the need for anticoagulation with IABP. In a trial with 153 patients requiring IABP at a single center between 2001 and 2004, there was a higher incidence of bleeding in the heparinized (14.1 %) vs. non-heparinized (2.4 %) group, but there was no difference in the incidence of limb ischemia [26]. As a result, there are no clear recommendations for the use of heparin to prevent thrombosis and embolization with IABP; however, heparin is used with most patients [27]. Although industry guidelines do not require anticoagulation with IABP, especially at a 1:1 ratio, heparin use with IABP in patients without contraindications, maintained >24 h, and with a lower ratio (e.g., 1:2) is thought to be reasonable [8]. However, we did not use heparin in the present case because of the tendency for large amounts of blood loss during hepatectomy.

The use of IABP during cardiac surgery has been well established; however, there are few studies regarding its use in non-cardiac surgery. To our knowledge, this is the first report of an IABP-assisted major hepatectomy. We believe that the perioperative use of IABP is beneficial for patients with severe cardiovascular disease who undergo non-cardiac surgery for various reasons.

## Consent

Written informed consent was obtained from the patient for publication of this case report and any accompanying images. A copy of the written consent is available for review by the Editor-in-Chief of this journal.

## Abbreviations

GSA: galactosyl human serum albumin
HCC: hepatocellular carcinoma
IABP: intra-aortic balloon pump
